# COVID-19 Infection Detection from Chest X-Ray Images Using Hybrid Social Group Optimization and Support Vector Classifier

**DOI:** 10.1007/s12559-021-09848-3

**Published:** 2021-03-04

**Authors:** Asu Kumar Singh, Anupam Kumar, Mufti Mahmud, M Shamim Kaiser, Akshat Kishore

**Affiliations:** 1 CSE Department, Maharaja Agrasen Institute of Technology, Delhi, India; 2 Department of Computer Science and Medical Technology Innovation Facility, Nottingham Trent University, Clifton, NG11 8NS Nottingham, UK; 3 Institute of Information Technology, Jahangirnagar University, Savar, 1342 Dhaka, Bangladesh

**Keywords:** Computer-aided detection system, Feature reduction, Evolutionary computing, Social group optimization

## Abstract

A novel strain of Coronavirus, identified as the Severe Acute Respiratory Syndrome-2 (SARS-CoV-2), outbroke in December 2019 causing the novel Corona Virus Disease (COVID-19). Since its emergence, the virus has spread rapidly and has been declared a global pandemic. As of the end of January 2021, there are almost 100 million cases worldwide with over 2 million confirmed deaths. Widespread testing is essential to reduce further spread of the disease, but due to a shortage of testing kits and limited supply, alternative testing methods are being evaluated. Recently researchers have found that chest X-Ray (CXR) images provide salient information about COVID-19. An intelligent system can help the radiologists to detect COVID-19 from these CXR images which can come in handy at remote locations in many developing nations. In this work, we propose a pipeline that uses CXR images to detect COVID-19 infection. The features from the CXR images were extracted and the relevant features were then selected using Hybrid Social Group Optimization algorithm. The selected features were then used to classify the CXR images using a number of classifiers. The proposed pipeline achieves a classification accuracy of 99.65% using support vector classifier, which outperforms other state-of-the-art deep learning algorithms for binary and multi-class classification.

## Introduction

**Fig. 1:**
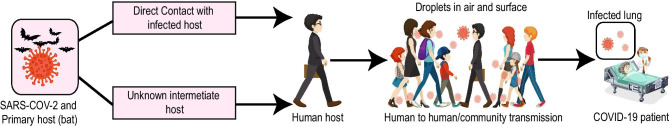
Block diagram showing SARS-CoV-2 transmission and spreading

In December 2019, China saw a sudden increase in pneumonia patients. Initially, the clear cause of this pneumonia remained shrouded in mystery. However, these were soon to be epidemiologically linked to the wet animal wholesale market [1, 2]. China alerted the World Health Organization (WHO) on the December 31 about the odd cases of pneumonia in one of its populous cities, Wuhan, Hubei Province. The novel virus behind all the unusual pneumonia cases was named SARS-CoV-2 by the WHO and was identified to be belonging to the coronavirus family, which caused Severe Acute Respiratory Syndrome (SARS-CoV) and Middle East Respiratory Syndrome (MERS-CoV) outbreaks. Corona-viruses infect birds and mammals including humans and can cause respiratory tract diseases of varying severity [3]. The first major outbreak of a coronavirus was the 2002-2004 SARS outbreak. The outbreak eventually was declared a global epidemic, with a total of 8000 reported cases and a 9% mortality rate worldwide [4]. The similarities between the viruses causing SARS-CoV and COVID-19 (SARS-CoV-2) are striking. Both viruses have 86% similar genome sequences that are analogous with SARS-like viruses found in bats, thereby, indicating that both viruses, transmitted from bats to humans at some point [5] as depicted in Fig. 1.

**Fig. 2:**
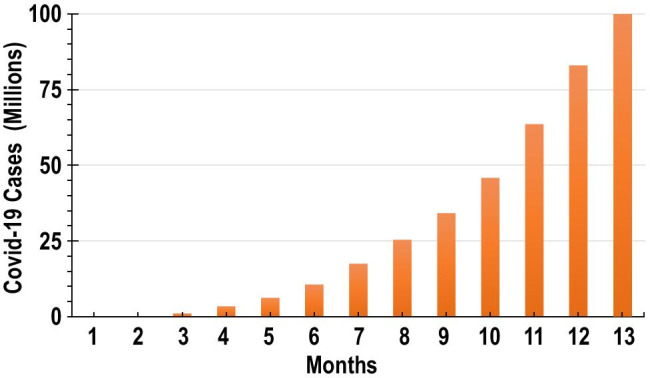
Number of COVID-19 cases across the globe during the last 13 months. The bar 13 is for January 2021 with data till January 24, 2021

The virus causing COVID-19 is highly transmittable and spreads mainly through coming in contact with respiratory droplets of an infected person. These droplets can penetrate the human body through inhalation or mouth [6]. As of the end of January 2021, according to statistics of the European Centre for Disease Prevention and Control, there have been almost 100 million confirmed cases worldwide with more than 2 million confirmed deaths as seen in Fig. 2. The mortality rates depend heavily on the age of the patients and prior medical conditions.

The prodrome of COVID-19 generally appears after an incubation period of 5-6 days. The most common symptoms of COVID-19 onset are: fever, cough, and fatigue, while in some cases, the patient may also have a headache, excess sputum, and diarrhea [7]. Particularly, in severe cases, this disease often causes pneumonia leading to oxygen deprivation, thereby damaging the body’s vital organs which results in fatal issues such as, kidney failure, heart failure, and other life-threatening complications. But a large number of reported cases show only mild symptoms and can be efficiently treated and managed. Studies reported that the patients with severe symptoms tend to be older in age and had multiple comorbidities such as cardiovascular, digestive or respiratory diseases [8].

The outbreak has caused a major stir worldwide and has led to strict lockdown and social distancing measures being implemented in every affected country. Even developed countries find it difficult to cope with the ongoing demand for intensive care units which are essential to support patients with severe cases of COVID-19. There is also a crisis for testing kits as the number of cases has started to pile up due to the virulence of the disease [9]. According to the latest guidelines, the testing method for COVID-19 is the reverse transcription polymerase chain reaction (RT-PCR). The test uses a respiratory sample that can be obtained by using a nasopharyngeal swab or sputum sample [10]. The RT-PCR test is reliable in the virulent period of the first week of infection but as time passes the virus might not appear in the throat as it completely moves down to the lungs and keeps multiplying. In this case, the coughed-up sputum samples are used to test [10].

Over the last few months, many researchers have been actively contributing towards methodological development for early COVID-19 detection and screening. This has been possible due to the recent developments of artificial intelligence (AI) and machine learning (ML)-based tools and techniques which have also been applied successfully to other tasks such as anomaly detection [12–14], biological data mining [15, 16], cyber security [17], disease detection [18–20], earthquake prediction [21], financial prediction [22], text analytics [23, 24] and urban planning [25]. Several AI and ML driven approaches have been developed to support COVID-19 [26] through analyzing lung images acquired by means of Computed Tomography (CT) [9], CXR [11][27], safeguarding workers in workplaces [28], identifying symptoms using fuzzy systems [29], and supporting hospitals using robots [30]. Many of the proposed solutions are based on computationally extensive deep learning (DL) models which are highly complex in nature and often have unreasonable computational costs.

The previously proposed DL models require extensive amounts of data in order to be trained, which could be difficult to obtain, in case of pandemic such as COVID-19. Hence, we require a robust solution that can work on small dataset and has comparable or higher accuracy than state-of-the-art DL models. Therefore, by using the inherent property of evolutionary algorithms of managing the smaller datasets with abridged computational complexity and relatively higher accuracy, we have developed a model which can be trained with smaller datasets and still yield respectable metrics.

In this paper, we propose a pipeline that uses CXR images to detect the COVID-19. In this pipeline the features from the radiographs are extracted and the relevant features are then selected using a modified Social Group Optimization (SGO) algorithm which we call Hybrid SGO algorithm (HSGO). The features selected using HSGO were then used in classifying the radiographs using the support vector classifier (SVC), K-nearest neighbor (KNN), decision tree (DT), and random forest (RF).

Towards the development of an improved, reliable and accurate pipeline, the current work can be summarized through the following highlights:

1. A model is proposed to classify COVID-19 infected patients from their CXR images.
2. The model is trained and tested on open dataset of COVID-19 infected CXR images sourced from Kaggle [31–33].
3. Hybrid Social Group Optimization algorithm is proposed which is used to select features from the CXR images.
4. The selected feature set is then used to classify the CXR images using various classifiers.
5. The proposed model achieves an accuracy of 99.65% using the Support Vector Classifier.
6. Deep learning counterparts had a maximum accuracy of 99.27%.
7. The proposed method has a higher accuracy and significantly lower training time compared to any available state-of-the-art deep learning algorithms.

The remainder of the article is organized as follows: Section 2 depicts the related works, the proposed method is described in Section 3, Section 4 details the experimentation process and results, followed by Section 5 which concludes the work and finally Section 6 highlights the possible future developments.

## Related Works

Since early 2020, several studies have been reported in the literature highlighting the shortcomings of RT-PCR testing, which includes a high false-negatives rate [34] and a short window for detection. In this public health emergency where the number of cases is increasing every day, the low sensitivity and the high rate of false-negatives means that patients will not be identified accurately, which hampers the chance of receiving appropriate treatment. The infected people, also run the risk of infecting others, thereby compounding the threat. In [35] the authors have conducted a thorough study on the spread and fatality rate of the pandemic, showing how fatality can be greatly reduced by minimizing the exposure of vulnerable groups to COVID-19.

The Computed Tomography (CT) scan of the chest is one of the most important methods in the diagnosis of pneumonia. Ai et al. showed a strong correlation between chest CT scans and RT-PCR test results in the identification of COVID-19 [36]. Apostolopoulos and Mpesiana [37] have proposed an automated COVID-19 detection system that utilizes CXR scans of the patient’s chest to diagnose the disease by using transfer learning with convolutional neural network (CNN). Some researchers have also shown CNN to be a great tool for identification of COVID-19 from CXR radiographs [38]. In [39], authors have suggested anti-aliased convolutional networks for detection of lung diseases. Also, a number of reported work have shown that chest CT scans are an important tool in COVID-19 diagnosis [9, 40–42].

Evolutionary algorithms have always played a critical role in medical image analysis by reducing the overall computation required by intensive DL or ML algorithms. Rundo et al. [43] surveyed the literature for the state-of-art of nature-inspired medical images analysis methods focusing on bio-medical data integration. Mostafa et al. [44] used whale optimization algorithm to segment liver from MRI scans by extracting features from different segments of the image with an accuracy of 97.5%. Woźniak et al. [45] used several bio-inspired algorithms to successfully detect pulmonary diseases from CXR images. The authors obtained the best accuracy of 82.22% using the particle swarm optimization algorithm. González-Patiño et al. [46] proposed a novel bio-inspired method based on bat algorithm for early identification of breast cancer by analyzing mammographic images with an accuracy of 97.42%. Hemanth and Anitha [47] proposed a modified genetic algorithm to classify brain images from four different classes with a final accuracy of 98%. Agrawal et al. [48] proposed a hybrid adaptive cuckoo search-squirrel search algorithm to analyze Brain MRI scans by obtaining optimal multi-level thresholds using maximization of the edge magnitude information. Wachs-Lopes et al. [49] discusses seven recent bio-inspired algorithms over multi-thresholding segmentation of medical images. The algorithms were tested for a range of values of non-extensivity parameter (‘q’), which is an essential parameter for Tsallis entropy. The firefly algorithm had the best performance and the Grey Wolf Optimizer with the fastest convergence.

There have been a number of studies on COVID-19 using CXR images, Ozturk et al. [50] proposed DarkCOVIDNet using CNN and DarkNet, 2-D convolution and Max Pooling for identification of COVID-19 from CXR images. The accuracy achieved by this model was 98.08%. Toğaçar et al. [51] used social mimic optimization along with SqueezeNet and MobileNetV2 deep learning architectures for the identification of COVID-19 from CXR images. The accuracy of this model was 99.27 (binary class) for covid-19 images, but the overall accuracy was 98.3% (COVID and Normal CXR images, multi-class). The accuracy of this model was indeed good, however, the method was not computationally efficient with two deep learning models. Panwar et al. [52] presented a model for fast identification of COVID-19 using the Vgg-16 and CNN deep learning-based model. The accuracy for the binary class for classifying COVID-19 images was 97.97% and normal image detection accuracy was 98.68%. Pereira et al. [53] used the pre-trained CNN network, the F1-score achieved 0.89, which is quite low as acknowledged by the author as well. Waheed et al. [54] proposed COVIDGAN an auxiliary classifier generative adversarial network (GAN), the method had a limitation, that very small dataset was used in order to train, then GAN was used to create synthetic dataset. The method achieved an accuracy between 85%–95% despite being biased towards the generated image dataset. Abdel-Basset et al. [55] proposed an improved marine predators algorithm, bettering the results obtained by other bio-inspired algorithm for COVID-19 detection with an overall fitness function value obtained 66.26. Oh et al. [56] proposed a method to segmentation of the lung images along with FC-DenseNet103 gave an overall accuracy of 88.9% for classification of COVID-19 images and 95% accuracy on classifying COVID-19 and Normal images. Vaid et al. [57] used simple pre-trained deep learning models and achieved an accuracy of 96.3% in classifying COVID-19 images. Mahmud et al. [58] proposed a multi-dilated CNN and achieved an accuracy of 97.4% for successfully detecting COVID-19 images. Moreover, Dey et al. [9] proposed SGO assisted Kapoor’s entropy to segment COVID-19 specific regions in CT scan images and used K-nearest neighbor method to classify. The method achieved an accuracy of 87.75%.

## Proposed Method

This work proposes a computer-aided diagnosis system that can automatically detect COVID-19 by using CXR radiographs of the patients. This system is expected to contribute in aiding doctors in the decision process, thereby reducing the time taken for accurate diagnosis and thus, hopefully, reducing the overall pressure on the medical staff as well. However, this should be noted that the has not been clinically validated like many other methods proposed in the literature and should not be used as an independent means to diagnose COVID-19.

The proposed pipeline consists of the following steps:

1. Preprocessing of the lung CXR radiographs;
2. Feature extraction from CXR radiographs;
3. Selection of relevant features using the HSGO algorithm;
4. Classification of the extracted features from the radiographs as healthy or infected.

**Fig. 3:**
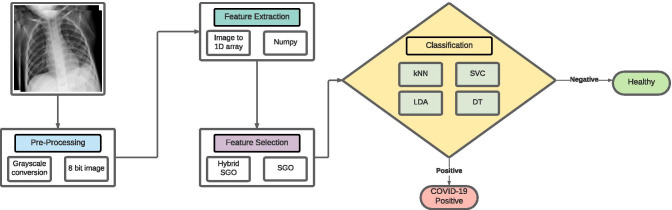
Flowchart of the proposed pipeline. It shows the main steps as: preprocessing, feature extraction, feature selection, and classification

The aforementioned pipeline is depicted in Fig. 3.

**Fig. 4:**
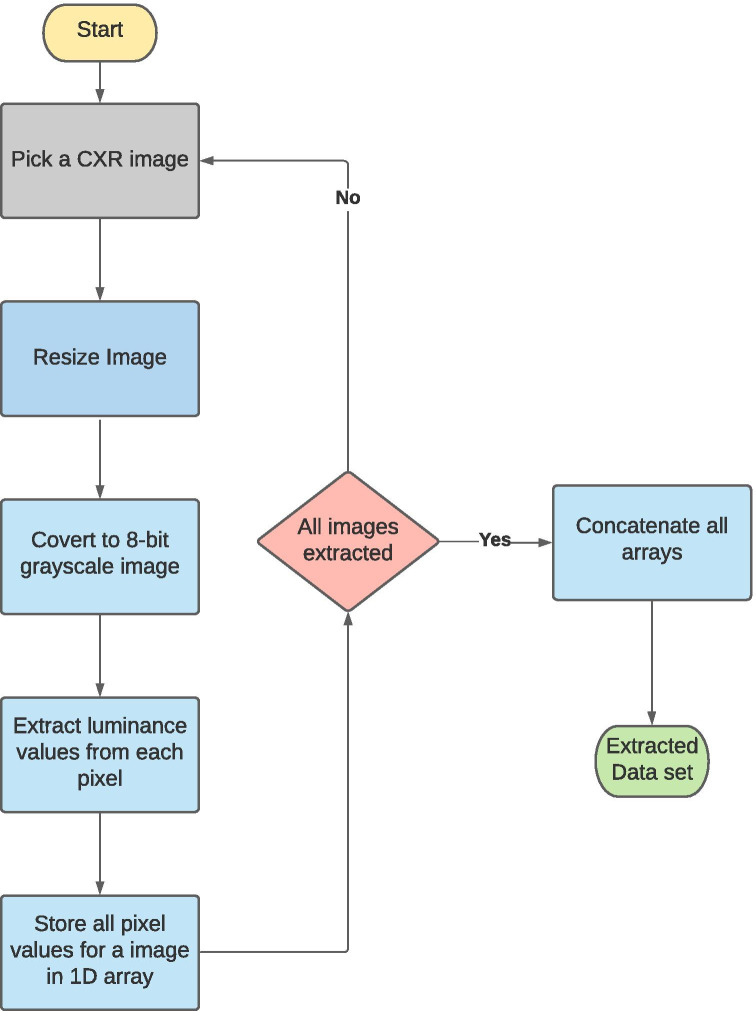
Preprocessing and feature extraction of lung CXR images

### Preprocessing and Feature Extraction

To detect COVID-19 infection through an automated classification method, features need to be extracted from the CXR images. In the first step, the given radiographs were resized to $100\times 100$ from their original size of $1024\times 1024$, which boosted computational speeds significantly and also reduced the extracted feature pool without compromising accuracy. All the features were extracted using Pillow 7.1.2 in grayscale mode [59] which extracts every pixel in the image by assigning a value according to the luminosity level of the pixel. The value is determined by the ITU-R 601-2 luma transform for every pixel in image. The lung CXR is first converted to a 8-bit image where every bit stores black and white levels thereby allowing 256 different shades. By doing this we can keep the subtle details and also minimize the extracted feature pool. Figure 4 depicts the preprocessing and extraction process in detail.

Other extraction methods like bi-level mode that converts a lung CXR to 1-bit image where value for every pixel is determined using Floyd-Steinberg dither to approximate luminosity levels, Palette mode which converts and gives 8 bit of data for each pixel allowing 256 colors and RGB mode that converts and stores the true color value of every pixel using $3\times 8$ bits were tested. All the aforementioned methods performed worse than the grayscale method. In case of Bi-level mode the final accuracy was considerably lower as many details required to classify an image were lost in conversion, whereas Palette and RGB mode both preserved the details but the number of features extracted was excessive for the problem at hand. Thus, using Grayscale mode the lung CXRs were converted and a total of 10,000 features were extracted from each one. The extracted feature pool was then subjected to feature selection, as described in subsequent sections.

### Feature Selection

In this section, we discuss feature selection from the extracted dataset using the proposed HSGO algorithm. This step is essential for reducing computational costs and also boosts accuracy. The feature selection method is based on the SGO algorithm, which is described below.

#### Social Group Optimization (SGO)

The SGO algorithm, proposed by Satapathy and Naik [60], is a meta-heuristic model based on population behavior that is inspired by the human group’s ability to solve a complex problem. It is based on the observation that a group’s problem-solving capability is better than an individual’s problem-solving ability as it exploits every member’s unique traits to solve a complex problem. Every person in the population is a candidate solution that has some knowledge about the solution to the given problem. The person with the best solution in the population imparts its knowledge to others, thereby increasing the overall knowledge of the entire population.

Mathematically, the SGO algorithm can be expressed as: let *N* be the number of persons in the population and each person is defined as $P_i = (x_{1}, x_{2}, x_{3},\cdots , x_{D})$ where *D* is the number of features which uniquely define a person. The features of every person need to be optimized to yield the best solution to the problem. For a given problem, the fitness function can be defined as $f_{i}$ where $0<i<N$ for every person in the population.

**Improving Phase** Every person acquires some knowledge from the best person by the following function: $x_{i,j}(new)=c\times x_{i,j}(old)+r\times (gbest_{j}-x_{i,j}(old))$ here, $gbest_{j}$ is the population’s best person, i.e., person with the best fitness value, *c* is the self-introspection factor, *r* is any random value $0<r<1$, and $x_{i,j}(new)$ is the *j*th feature for the *i*th person in the population which is accepted if it gives better fitness value.

**Acquiring Phase** Every person in the population learns from random persons in the population and from the population’s best person by the following function: $x_{i,j}(new)=x_{i,j}(old)+r1\times (x_{r,j}-x_{i,j})+r2\times (gbest_{j}-x_{i,j})$ where $x_{i,j}(old)$ is the initial value of the *j*th feature for the *i*th person in the population, $gbest_{j}$ is the population’s best person, i.e., person with the best fitness value, *c* is the self-introspection factor, *r*1 and *r*2 are any random values $0<r1,\ r2<1$, and $x_{i,j}(new)$ is the *j*th feature for the *i*th person in the population which is accepted if it gives better fitness value.

**Table 1:** Comparison of Feature Selection Methods

Selection Method	Features Selected	Accuracy (%)	Classifier Used
Hybrid SGO	116	99.65	SVC
SGO	254	99.31	SVC
KPCA	500	99.31	SVC
PCA	511	99.31	SVC

**Figure:**
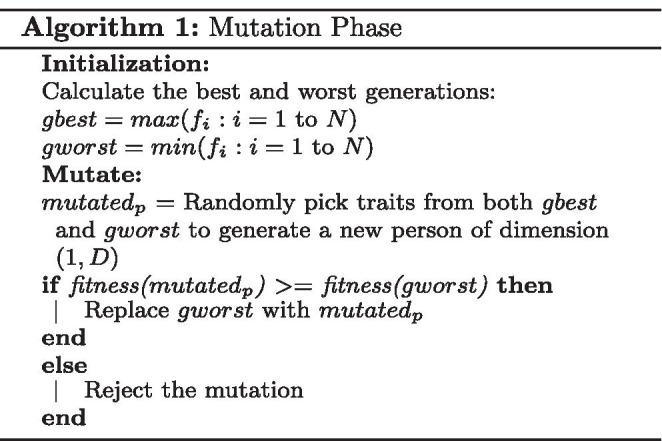


#### Hybrid Social Group Optimization (HSGO)

The HSGO algorithm is an improved version of the SGO algorithm, which has been developed to select optimal features from a feature pool. By following a wrapper-based approach, HSGO gives the optimal feature set in considerably fewer iterations. Theoretically, both SGO and HSGO should eventually give the optimal feature set but in our experiments HSGO outperforms SGO and other conventional feature selection methods by selecting a markedly smaller feature set which also gives a noticeably better accuracy. Table 1 compares HSGO with SGO and other conventional methods. This improvement can be attributed to addition of a random factor which helps HSGO to overcome local minima or maxima traps, when compared to the traditional SGO algorithm. The SGO algorithm when used for feature selection, tends to yield the same results after a few generations of notable improvement in the population. The effect of this random factor is evident especially when the number of features in the dataset are high ($>1000$). The random factor is obtained by introducing a new step called the Mutation phase, this phase is explained in Algorithm 1. Algorithm 2 shows the proposed HSGO algorithm which can be broadly divided to the following steps.

**Figure:**
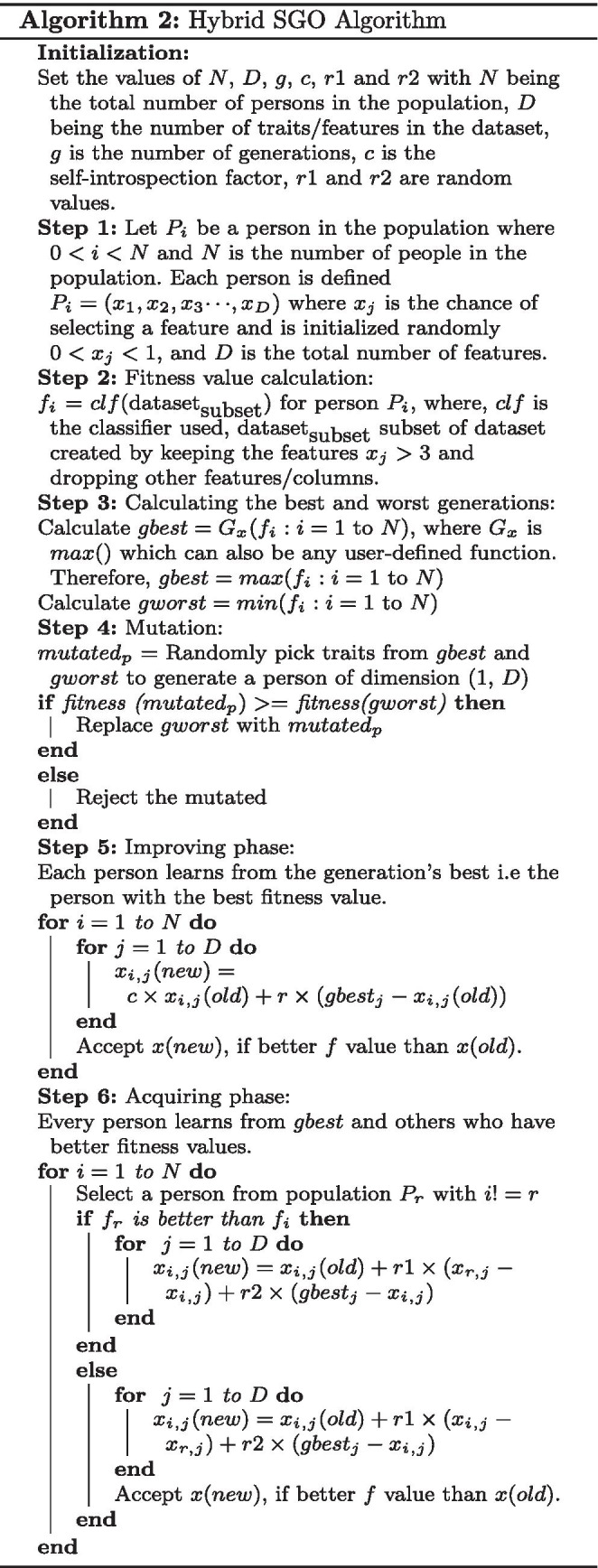


**Fig. 5:**
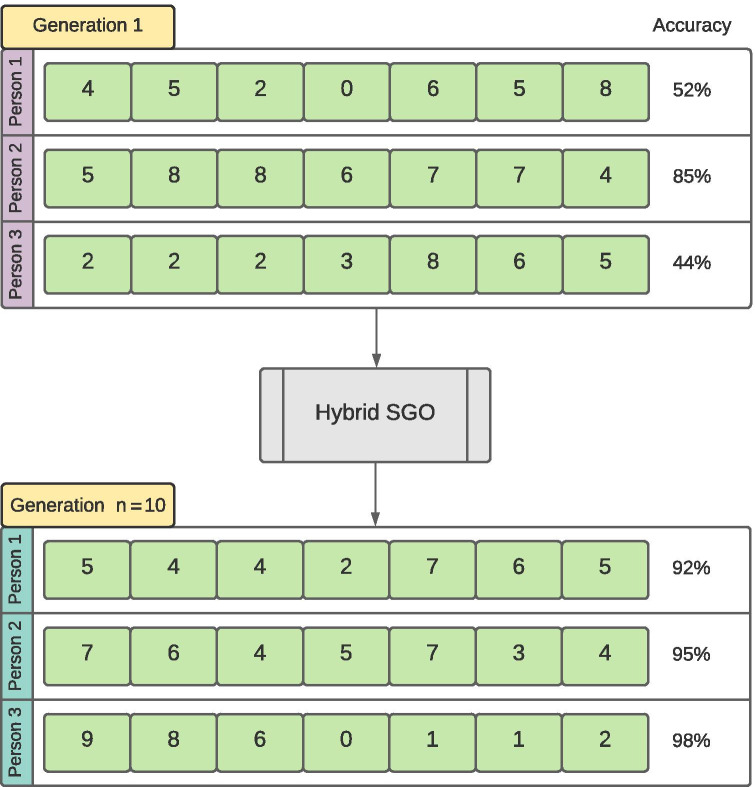
Initialization and generational improvement provided by the hybrid SGO algorithm

**Initialization function** The initialization function initializes each person in the population by making a random array of integer values ranging from 0 to 9 with dimensions (*N*, *D*) where *N* is the number of people in population and *D* is total extracted features. The range of 0 to 9 was chosen to allow enough randomness in features of every person in the population while at the same time making it much easier to trace the learning steps. The initialization and improvement process is shown pictorially in Fig. 5.

**Fitness function** The fitness function used here is the classifier which will be used on the optimal subset of features. It gives the fitness and the accuracy of the current subset of the dataset by assigning fitness values to each person in the population after every generation.

$$\begin{aligned} f_{i} = classifier(P_i) \end{aligned}$$

where, classifier is the classifier being used, $P_{i\ }$ is the *i*th person in the population, and $f_{i}$ is the calculated fitness.

**Mutation Phase** The purpose of the mutation phase is to facilitate continuous improvement in the population by constantly introducing a new person whose traits are notably different from the persons currently in the population. Algorithm 1 shows the mutation phase of the process.

A subset of the dataset is created for each person in population, every generation, by selecting only the corresponding columns of the dataset where the persons feature value is greater than 3. Initially, we selected the columns from dataset which had corresponding feature value of 5 or more, but it was changed to 3 to get a wide variety of feature subsets in the initial population. The subset created is then used to calculate accuracy using appropriate classifiers and is gradually improved with every generation to give the final selection of features that give the best accuracy and minimum computational cost.

The HSGO algorithm selected 116 features out of 10,000 raw features extracted from lung CXR images. In other words, the algorithm selected only 1.16% features from the extracted feature set, therefore, filtering out 98.84% of insignificant features. Table 1 shows the comparison of various feature selection methods used, accuracy obtained using the feature subset and the classifier used.

## Experimentation and Results

In this section, we discuss the implementation of the HSGO algorithm. It comprises of setup specifications along with the various parameters used in the algorithm and classifiers.

### Dataset

**Fig. 6:**
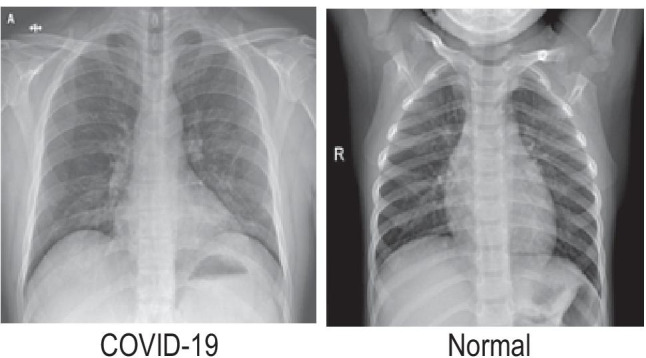
Example chest X-ray images from the dateset – COVID-19 (left), and normal (right)

The dataset used in this work was obtained from the Kaggle repository ”COVID-19 Radiography Database”. The database from this repository consisted of 219 COVID-19 positive images, 1341 normal images, and 1345 viral pneumonia images. [31–33]. Adding 152 more COVID-19 positive images from other similar sources, the total number of images for this class was increased to 371. Our experiments were performed using CXR radiographs of positive COVID-19 cases and normal images from the dataset. All the images were resized from 1024$\times$1024 to 100$\times$100 pixels, which substantially reduces the feature pool extracted and boosts computational speed. All the results quoted in the work were achieved using the original dataset. Representative radiographs for both COVID-19 infected patients and normal cases are given in Fig. 6, respectively. As from the individual image numbers from different classes it can be seen that the dataset is imbalanced, we created 3 subsets from the full dataset for evaluation. The split ratio between the COVID-19 and non-COVID-19 CXR images were changed by either increasing or decreasing the non-COVID-19 CXR images to make the subsets to contain 30% COVID-19–70% non-COVID-19 images, 50% COVID-19–50% non-COVID-19 images, and the full dataset containing all COVID-19 and non-COVID-19 CXR images. It should be noted here that, this split was solely for the sake of evaluation, the final model was trained on the original dataset.

### Experimental Setup

All tests were performed on Google Colaboratory, allowing execution of python code in browser, on Google Cloud Servers running on a Intel(R) Xeon(R) CPU @ 2.30 GHz, having 20 physical cores paired with a K80 GPU and RAM capacity up to 36 GB. A maximum of 108 GB of disk space was available, out of which 33 GB was used for the experiments. Pillow library, a fork of Python Imaging Library was used for extracting features from the lung CXRs. All the algorithms were developed using Python 3.6.7 as the language and NumPy, Pandas, and Scikit-learn libraries.

### Input Parameters

The parameters used in the HSGO algorithm are shown in Table 2 and the parameters of the various classifiers are reported in Table 3.

Among the parameters of the HSGO algorithm, the *N* and *D* are constant values which are chosen as per the problem requirements. The self-introspection factor *c* used in the improving phase controls the knowledge gained from the generation’s best at each iteration and can be set from $0< c < 1$. If the value of c is too low, the learning steps are too flat, which can be beneficial in some cases whereas higher values of *c* $(>0.8)$ results in steep learning steps and is generally unstable. In our experiments $c=0.7$ was used which yields a respectable learning rate without compromising stability. *r*1 and *r*2 are independent random numbers which affect the stochastic nature of the algorithm. The value of *r*1 and *r*2 can be $0< r1, r2 < 1$. We have used $r1=0.6$ and $r2=0.8$ in our experiments to achieve the stated results. All the parameters used in classifiers were obtained by the process of hyper parameter tuning using Grid Search algorithm. The algorithm determines the best parameters by methodically checking the specified classifier against a set of possible parameters thereby ensuring best metrics.

**Table 2:** Parameters for the HSGO Algorithm

Parameter	Value	Description
*N*	5	No. of Persons
*D*	10,000	No. of features
*r*1	0.6	Random value r1
*r*2	0.8	Random value r2
*c*	0.7	Self-Introspection factor

**Table 3:** Parameters for Machine Learning Algorithms

Classifier	Parameters Used
KNN	n-neighbors = 3, metric = manhattan
DT	criterion = gini, splitter = best
RF	criterion = gini, max-depth = 2, n-estimators = 100
L-SVC	penalty = ’l2’, loss = ’squaredhinge’, maxiter = 1000
SVC	C = 1.0, kernel = ’rbf’, degree = 3, gamma = ’scale’, maxiter = −1
PCA	n_components = 0.98, random_state = None
KPCA	kernel = ’linear’, n_components = 500

**Fig. 7:**
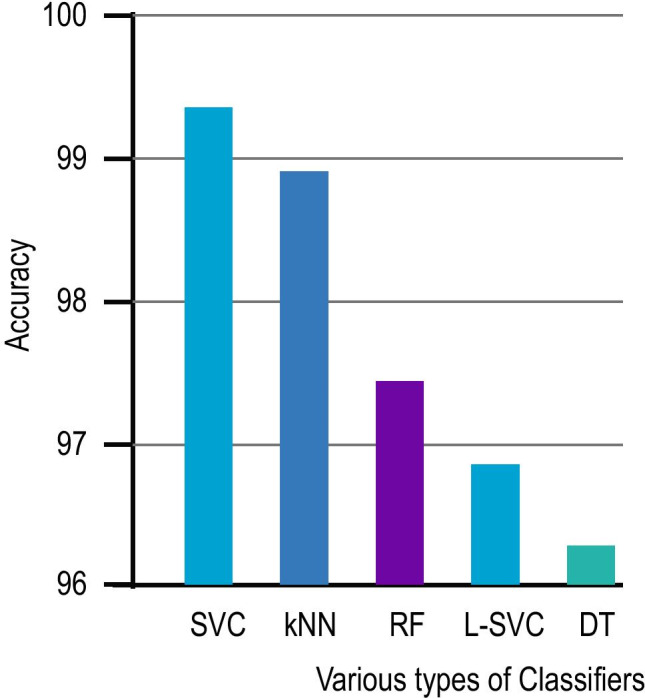
Classification accuracy of various classifiers

**Table 4:** Performance comparison of different classifiers

Class.	Prec.	Sens.	F1S
KNN	0.9896	0.9897	0.9897
DT	0.9489	0.9591	0.9611
RF	0.9758	0.9761	0.9759
L-SVC	0.993	0.993	0.993
SVC	0.9966	0.9965	0.9965

*Class* Classifier, *Prec* Precision, *Sens* Sensitivity, *F1S* F1 score

**Fig. 8:**
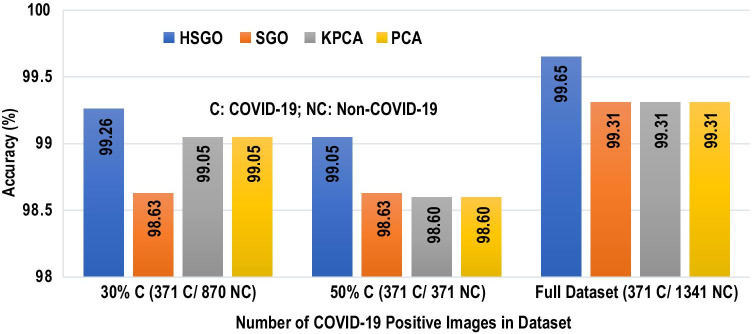
Effect of number of images from COVID-19 and non-COVID-19 classes on classification performance

### Performance

The proposed pipeline with HSGO algorithm was applied on the complete dataset (i.e., 371 COVID-19 positive CXR images and 1341 non-COVID-19 CXR images) and the classification of the features was performed by five different methods, namely, KNN, DT, RF, L-SVC, and SVC. As shown in Fig. 7, the best accuracy of 99.65% was obtained with the SVC classifier and this was consistent with the F1-score obtained for the SVC method reported in Table 4.

To assess the effect of the number of COVID-19 images on the accuracy, the proposed pipeline with HSGO algorithm and SVC classifier was applied on three different subsets of data (see "Dataset" section for details). It was observed that the highest accuracy of 99.65% was obtained when the complete dataset was used. Also, comparing with other optimization algorithms, as shown in Fig. 8, this highest accuracy was obtained with the HSGO algorithm on the complete dataset in comparison to other methods (such as SGO, KPCA, and PCA) and dataset size. On the same dataset the SGO, KPCA, and PCA methods provided an accuracy of 99.31%. When the number of COVID-19 positive CXR images were 30% and 50% and the non-COVID-19 images were 70% and 50%, respectively, the proposed HSGO algorithm outperformed all other methods. In case of the 3:7 dataset, the highest accuracy was 99.05% with HSGO and in case of the 1:1 dataset, the highest accuracy was 99.26%.

Evidently, the performance of the pipeline is consistent even after altering the composition of the dataset showing the classification accuracy of minority class, i.e., COVID-19 is maintained. Based on the above discussions, it can be claimed that the proposed HSGO-based pipeline, in combination with the SVC, is capable of detecting COVID-19 from CXR radiographs remarkably well.

**Fig. 9:**
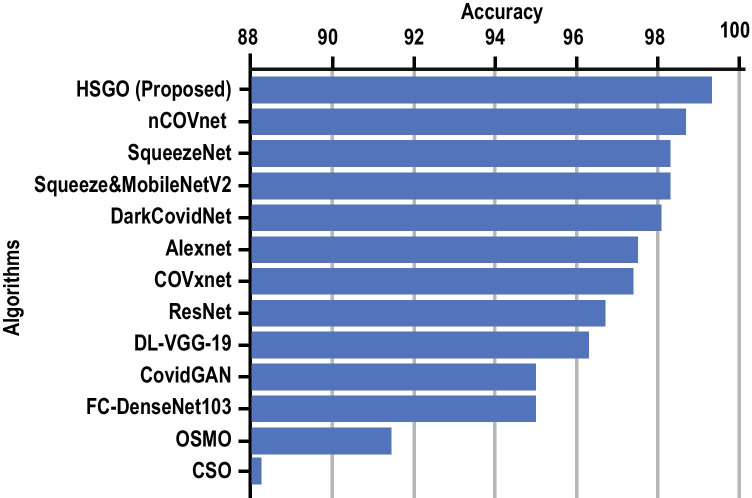
Accuracy comparison with other Deep Learning and other Bio-Inspired Algorithms. References: nCOVnet [52], SqueezeNet [38], Squeeze&MobileNetV2 [51], DarkCOVIDNet [50], Alexnet [38], COVxnet [58], ResNet [38], DL-VGG-19 [57], COVIDGAN [54], FC-DenseNet103 [56], OSMO [61], and CSO [62]

### Benchmarking with State-of-the-Art Deep Learning Models and Evolutionary Algorithms

The results obtained with the HGSO algorithm were also compared with other similar bio-inspired algorithms namely Chaotic Crow Search Optimization algorithm (CSO) and Spider-Monkey Optimization algorithm (OSMO). These algorithms are tweaked versions of the original crow search algorithm and spider monkey optimization algorithm, respectively. The CSO algorithm is a meta-heuristic optimizer which takes inspiration from a crow’s searching methods to hide extra food and its retrieval when needed [61]. On the other hand, the OSMO algorithm is a swarm intelligence technique [62] that relies on the collective intelligence of a group to solve the problem at hand. Both algorithms were used as a feature selection method for the same dataset and the selected feature set was then used with every classifier. The maximum accuracy obtained were saved and plotted. As seen in Fig. 9, the proposed method performs better than aforementioned meta-heuristics.

The authors in [38] used four pre-trained deep CNN architectures, namely, AlexNet, ResNet18, SqueezeNet and DenseNet201 to classify normal and COVID-19 infected radiographs with an accuracy as high as 98.3% using the SqueezeNet. A comparison of the accuracy of our model against the state-of-the-art DL (as discussed in section 2) and other optimized algorithms have been depicted in the bar graph in Fig. 9, Evidently the proposed method outperforms every other method with 99.65% accuracy using the SVC classifier.

## Conclusion

The ongoing global pandemic due to the COVID-19 outbreak has led to a global crisis worldwide. Even developed countries are struggling to cope with the demand for medical supplies and testing kits. The shortage of testing kits especially hampers efforts to stop the spread of the disease, as many cases go undetected, which may lead to even more infections of COVID-19. Early diagnosis of the disease is essential to stop further spread and reduce mortality rates. The proposed solution of a computer-aided diagnosis system, uses CXR radiographs of patients to automatically predict COVID-19. Our experimental results show that the model with SVC yields the highest accuracy of 99.65% among all classifiers. The proposed pipeline, due to its high accuracy and precision, can be used to develop mobile applications that can be an aid in early diagnosis of COVID-19 for medical practitioners.

## Future Scope

 The proposed pipeline’s accuracy can be further improved. For instance, the HSGO model can be made more accurate by increasing the number of COVID-19 chest CXR images. The effects of numerous parameters used in HSGO can be studied in depth on different datasets to unravel how they all come together and affect the final results. Alternative feature extraction methods can be experimented with the proposed pipeline to further enhance the end result. The Mutation phase of the HSGO can also be adjusted according to the problem at hand, thereby allowing widespread applications in countless real-world problems.

## Data Availability

The code can be accessed via the following GitHub repository: https://github.com/Enixes/Hybrid-Social-Group-Optimization-algorithm.
